# Application of Computational Chemical Shift Prediction Techniques to the Cereoanhydride Structure Problem—Carboxylate Complications

**DOI:** 10.3390/md15060171

**Published:** 2017-06-12

**Authors:** Carla M. Saunders, Dean J. Tantillo

**Affiliations:** Department of Chemistry, University of California—Davis, 1 Shields Avenue, Davis, CA 95616, USA; cmsaunders@ucdavis.edu

**Keywords:** computational NMR, natural products, chemical shifts

## Abstract

Despite the vast array of techniques available to modern-day chemists, structural misassignments still occur. These misassignments are often only realized upon attempted synthesis, when the spectra of synthesized products do not match previously reported spectra. This was the case with marine natural product cereoanhydride. The originally proposed 7-membered ring anhydride (**1**) was shown to be incorrect, although a likely precursor to the correct structure (**2**) in both its laboratory synthesis and biosynthesis. Herein, in addition to showing how NMR computations could have been used to arrive at the correct structure, we show that the conversion of **1** to **2** is indeed energetically viable, and we highlight complications in predicting NMR chemical shifts for molecules with acidic protons.

## 1. Introduction

The isolation and structural assignment of natural products remains a very active field, continuing to lead to the discovery of many important compounds. Fungi in particular are known to produce secondary metabolites with unique structures, often useful in drug discovery [1,2,3]. Unfortunately, the structures of many natural products have been misassigned initially, leading to frustration for chemists following up on these structures [4,5]. Computational chemistry, in particular NMR prediction using quantum chemical methods, can be used as a tool to facilitate confirmation, assignment, and reassignment of natural product structures [6,7,8,9,10] and therefore can be used to focus laboratory experiments on correct structures. While computational methods for predicting ^1^H and ^13^C chemical shifts are well established [6,7,8,9,10], these methods do not work without fail. We are particularly interested in cases where readily exchangeable protons affect ^13^C chemical shifts, e.g., chemical shifts for carbons near to amines or carboxylic acids [11]. In such systems, NMR spectra are pH-sensitive, and such molecules tend to oligomerize, making spectral prediction particularly difficult [6,12,13,14]. Here, we describe a case study that highlights these difficulties and first steps to address them.

**Figure 1:**
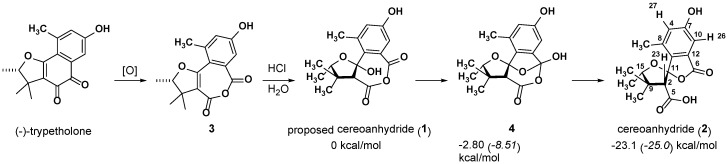
Structures investigated and their relative energies in kcal/mol: B3LYP/6-31+G(d,p) free energies in normal text; SMD(CH_3_OH)-mPW1PW91/6-311+G(2d,p)//B3LYP/6-31+G(d,p) energies in italics.

Interested in fungal natural products, Wolfender, König, and co-workers isolated the fungus *Coniothyrium cereal* from green algae in the Baltic Sea [15]. After cultivating this fungus in saline, several interesting compounds were isolated. The structure **1**, dubbed “cereoanhydride”, was assigned to one of these compounds on the basis of data obtained from MS, IR, and multiple NMR techniques. The structure proposed was also subjected to computation, but only conformational searches using molecular mechanics to confirm that the geometry was consistent with an observed NOE interaction. It was proposed that (−)-trypetholone, a previously isolated natural product, was a precursor to cereoanhydride (Figure 1). Cereoanhydride was found to selectively inhibit human leukocyte elastase, a protease that causes tissue damage and plays a crucial role in many inflammatory diseases [16,17]. 

Recently, cereoanhydride was synthesized by Hu and co-workers [18,19]. In order to follow the biosynthetic route proposed by Wolfender and König, (±)-trypetholone was first synthesized in seven steps, and then exposed to Baeyer–Villiger oxidation conditions to produce **3**. The NMR spectra of the product formed on exposing **3** to acid matched the NMR spectra reported for cereoanhydride. However, an X-ray structure of the isolated material corresponded to structure **2** instead, suggesting that the structure of cereoanhydride was initially misassigned. However, Hu and coworkers suggested that **2** was formed via **1** (the originally assigned structure) and intermediate **4**, as shown in Figure 1.

Below we describe quantum chemical computations on ^1^H and ^13^C NMR chemical shifts for structures **1** and **2**, and energetics for the **1** → **4** → **2** conversion (all computed using density functional theory [DFT]; see Methods section for details). It was our goal to determine whether the correct structure of cereoanhydride could have been assigned on the basis of the originally reported NMR data in combination with quantum chemical computations. Our results demonstrate once again [6,7,8,9,10] the utility of NMR computations in the assignment of natural product structures, and highlight the complications of predicting chemical shifts for carboxylic acid-containing molecules.

## 2. Results and Discussion

### 2.1. ***1*** as a Precursor to ***2***

The structures and energies of **1**, **2**, and **4** were calculated using B3LYP/6-31+G(d,p) in the gas phase. The energies of the lowest energy conformers of each were then computed using SMD(CH_3_OH)-mPW1PW91/6-311+G(2d,p). The proposed **1** → **4** → **2** pathway was found to be downhill for each step, and highly exergonic/exothermic overall. Intermediate **4** was found to be 2.8 kcal/mol lower in energy than **1** with B3LYP, and 8.5 kcal/mol lower with mPW91PW91, while **2** was found to be 23.1 kcal/mol lower in energy than **1** with B3LYP, and 25.0 kcal/mol lower with mPW1PW91. Thus, the **1** → **4** → **2** pathway is not only predicted to be thermodynamically favorable, but also effectively irreversible. The computed energy differences can be ascribed to a combination of effects. For example, the 7-membered ring of **1** is clearly strained, as evidenced by bond angles that deviate significantly from ideality, e.g., the anhydride C–O–C angle is large (132°). The anhydride carbonyls also are not coplanar, leading to decreased conjugation. Product **2** does not suffer from these issues.

Because dispersion is known to affect some systems, the above structures and energies were also calculated using B3LYP-D3/6-31+G(d,p) [20,21]. Again, the energies of the lowest energy conformers of each were then computed using SMD(CH_3_OH)-mPW1PW91/6-311+G(2d,p). These results agreed with the results obtained without dispersion correction, suggesting that dispersion plays a small role in this system. Using B3LYP-D3, intermediate **4** was found to be 1.6 kcal/mol lower in energy than **1**, while **2** was found to be 21.9 kcal/mol lower in energy than **1**. 

### 2.2. NMR Chemical Shifts of ***1*** vs. ***2***

**Figure 2:**
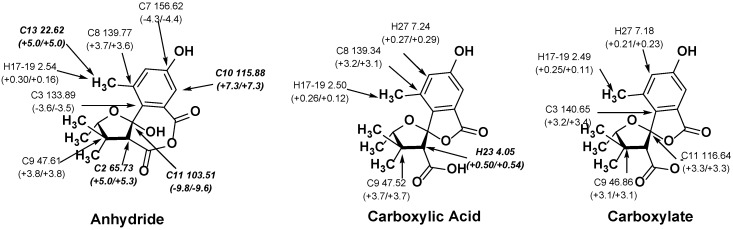
Deviation between experimental and computed shift (ppm) in **1**, **2**, and deprotonated **2**. Deviations outside of acceptable values are indicated with bold and italics. Deviations below 0.2 ppm (^1^H) and 3.0 ppm (^13^C) are excluded for clarity.

^1^H and ^13^C NMR chemical shifts for the anhydride (**1**) and the acid (**2**) were calculated using DFT. Each compound was found to have multiple accessible conformers, so Boltzmann weighted averages of these were used to arrive at chemical shift predictions. All conformers within 3 kcal/mol of the lowest energy conformer were considered. The chemical shifts for each conformer were calculated using mPW1PW91/6-311+G(2d,p) (a linear scaling approach [22,23] was used; see Methods section for details). The Boltzmann-weighted average shifts, their maximum absolute deviations (MAX) and mean absolute deviations (MAD) between computed and experimental chemical shifts are shown in Table 1 and Figure 2 (NMR results using B3LYP-D3 geometries were almost identical to those obtained with B3LYP; see Supporting Information). Although the MAD values for the structure of **2** are smaller than those for **1**, the large differences between computation and experiment for several specific chemical shifts suggest that **2** is not the correct structure. In particular, H23, the proton α to the acid, is unacceptably far downfield, with a deviation of 0.54 ppm.

An alternative method of determining which computed structure best matches experimental NMR data is DP4 analysis, developed by Goodman and co-workers [22]. This analysis provides the percent likelihood that each compound is the correct match with experimental data on a shift-by-shift basis. When this analysis for the studied system was considered, a picture similar to that from MAD analysis was reached. If only ^13^C shifts are considered, **2** is the most likely structure (81.7% probability). However, consideration of ^1^H shifts alone, or both ^13^C and ^1^H shifts together, suggests that deprotonated **2** is a better match, yielding 61.9% and 86.4% probabilities, respectively.

**Figure 3:**
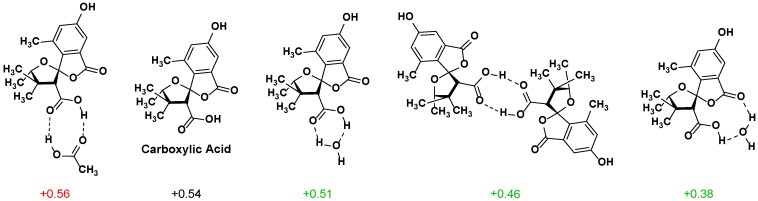
Deviation between experimental and computed shift (ppm) of α proton in various H-bonding models compared to the corresponding shift of **2**.

Given the match between the experimental shifts and those computed for the carboxylate form of **2**, we hypothesized that deprotonated **2** may be present to some extent. On the basis of p*K*_a_ values, however, this would be a very small amount. Another option is that hydrogen bonding to the carboxylic acid form would have a similar, although smaller, effect. To test this conjecture, several H-bonded complexes were modeled, including a dimer system, as carboxylic acids are known to dimerize in solution [23] (Figure 3). Although the deviations for the α proton improved for most of these, none were within our accepted values. A model of a carboxylic acid–carboxylate complex yielded acceptably close values for all proton and carbon signals. While we do not know how much carboxylate might actually be present in the experimental NMR sample, we can say that the observed chemical shifts are consistent with the carboxylate being present rather than absent, at least in the absence of an alternative explanation that has not yet come to light.

**Figure 4:**
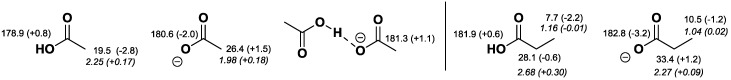
Computed shift and absolute shift deviation from experimental shift (ppm) for ^13^C NMR (normal text) and ^1^H NMR (italics) of acetic acid, acetate, and acetate/acetic acid complex (**left**) and propanoic acid and its conjugate base (**right**).

A large shift difference between the carbonyl carbon of the acid and the carboxylate was not observed computationally. While some systems do have large shift changes at this position, often the shift in ^13^C signal from acid to carboxylate is within the error of our calculations (usually <5 ppm) [14,24,25]. In general, NMR calculations underestimate the magnitude of this ^13^C shift. In addition, our calculations for H-bonding networks appear to be approximately as accurate as for the corresponding acids. A small system, based on acetic acid, demonstrating this is shown in Figure 4 (left). The calculated carbonyl carbon chemical shift for the acetic acid has the smallest deviation from the experiment, followed by the hydrogen-bonded acetate/acetic acid complex, and finally the acetate, although all deviations are small. Small deviations between experiment and theory are also observed for the α- and β-carbon shifts (all <3 ppm) and for the α- and β-proton shifts (all ≤0.3 ppm). Similar results are obtained for propanoic acid (Figure 4, right). The results obtained for these model systems give us confidence in the accuracy of the computed shifts for cereoanhydride.

**Figure 5:**
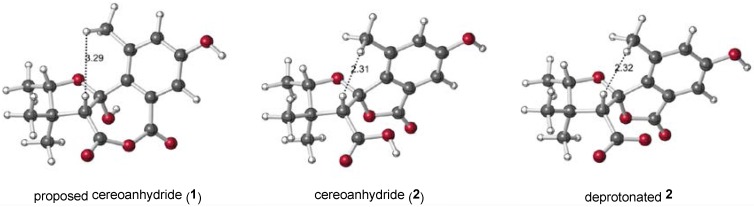
Computed structures of the lowest energy conformers of **1**, **2**, and deprotonated **2**, with distance corresponding to the NOE interaction discussed in the text shown in Å.

NOE data provided with the initial structural assignment show a correlation between the groups indicated in Figure 5 [15]. Wolfender, König, and co-workers computed a distribution of conformers for the proposed anhydride structure and found that the methyl and methine groups were close enough for this NOE interaction (although the exact distance found was not specified). NOE signals are usually only seen for distances of ≤5 Å. For the lowest energy conformers computed with DFT (Figure 5), the methyl-methine distance in the anhydride (3.29 Å), the acid (2.31 Å), and the carboxylate (2.32 Å) are all short enough that NOEs could be observed. While this structural argument is not definitive, it is consistent with the chemical shift argument for (deprotonated) **2** described above.

## 3. Conclusions

The results of DFT-based NMR chemical shift computations are consistent with the structural assignment of Hu and coworkers. In addition, the conversion of the originally proposed structure of cereoanhydride (**1**) to the revised structure (**2**) is predicted to be a very exergonic process. In short, we have shown here that NMR computations could have distinguished between structures **1** and **2**. In addition, this study highlights the complications that arise when multiple protonation states and hydrogen-bonded complexes of polar molecules may be present in experimental NMR samples.

## 4. Methods

Quantum chemical calculations were carried out using *Gaussian09* [26,27]. Structural optimizations and frequency calculations were performed with B3LYP/6-31+G(d,p) in the gas phase [28,29]. Additionally, these calculations were repeated with B3LYP-D3/6-31+G(d,p) [20]. NMR calculations (GIAO) for the cereoanhydride systems were performed with mPW1PW19/6-311+G(2d,p) using the SMD continuum solvent model for methanol [13,30,31,32,33,34,35,36,37]. NMR calculations (GIAO) for the acetic acid and propanoic acid systems were performed with mPW1PW19/6-311+G(2d,p) using the SMD continuum solvent model for water [13,30,31,32,33,34,35,36,37]. Isotropic shielding values were obtained and scaled to arrive at chemical shifts [6,38,39]. For methyl groups, the average of all three scaled ^1^H shifts was used to compare to experimental data. For each compound, a systematic conformational search was performed using *Spartan ‘10* [40]. Each conformer found was optimized with B3LYP/6-31+G(d,p), and NMR calculations were performed only on conformers within 3 kcal/mol of the lowest energy conformer. These approaches are well precedented for modeling organic reactions [41] (tests with a method known to better treat dispersion are described in the Supplementary Materials) and for predicting NMR chemical shifts [6,39,41].

## Figures and Tables

**Table 1:** Calculated and experimental chemical shifts for **1**, **2**, and deprotonated **2** (carboxylate), with deviations shown (largest deviations are shown in bold italics). ^1^

Atom Label	Exp. δ	Anhydride (1) δ	Abs. Dev.	Acid (2) δ	Abs. Dev.	Carboxylate δ	Abs. Dev.
C6	170.7	168.7	2.0	171.4	0.7	172.2	1.5
C5	170.4	168.9	1.5	172.5	2.1	172.4	2.0
C7	161.0	156.6	4.4	159.1	1.9	158.4	2.6
C3	137.4	133.9	3.5	138.2	0.8	140.7	3.3
C8	136.2	139.8	3.6	139.4	3.2	138.2	2.0
C12	131.0	132.7	1.7	129.8	1.2	131.2	0.2
C4	124.7	123.5	1.2	124.6	0.1	123.3	1.4
C11	113.1	***103.5***	***9.6***	112.0	1.1	116.6	3.5
C10	108.6	***115.9***	***7.3***	108.1	0.5	107.6	1.0
C15	87.4	85.6	1.8	86.6	0.8	85.9	1.5
C2	60.4	***65.7***	***5.3***	59.0	1.4	61.6	1.2
C9	43.8	47.6	3.8	47.6	3.8	46.9	3.1
C16	26.1	24.8	1.3	24.4	1.7	26.0	0.1
C13	17.6	***22.6***	***5.0***	17.9	0.3	17.8	0.2
C1	17.2	16.0	1.2	16.1	1.1	16.7	0.5
C14	14.9	14.6	0.3	14.0	0.9	15.1	0.2
	MAD ^2^		3.3		1.3		1.5
	MAX ^3^		***9.6***		3.8		3.5
H27	6.95	7.11	0.16	*7.24*	*0.29*	7.18	0.23
H26	6.93	7.10	0.17	7.11	0.19	7.05	0.12
H29	4.21	4.17	0.04	4.38	0.17	4.23	0.02
H23	3.51	3.58	0.07	***4.05***	***0.54***	3.66	0.15
H17-19	2.28	2.54	0.16	2.64	0.26	2.49	0.11
H28,31,33	1.36	1.31	0.05	1.38	0.02	1.47	0.11
H25,30,32	1.28	1.31	0.03	1.31	0.03	1.29	0.01
H20-22	1.28	1.37	0.09	1.40	0.12	1.42	0.14
	MAD ^2^		0.10		0.20		0.11
	MAX ^3^		0.17		***0.54***		0.23

^1^ Protons not seen in the experimental NMRs are not included here (OH’s and acid H). ^2^ Mean absolute deviation. ^3^ Maximum absolute deviation. Deviations of <5 ppm (^13^C) and <0.3 ppm (^1^H) are generally considered acceptable [6,7,8,9,10].

## Supplementary Materials

The following are available online at www.mdpi.com/1660-3397/15/6/171/s1. Anhydride lowest energy conformers and NMR values; intermediate **4** lowest energy conformers and NMR values; acid lowest energy conformers and NMR values; deprotonated acid lowest energy conformers and NMR values; NMR values for H-bonding systems; results from B3LYP-D3 calculations; NMR values for acetic acid- and propanoic acid-based systems; full Gaussian references; atomic coordinated for calculated structures.
