# Supplementary material for: Application of Computational Chemical Shift Prediction Techniques to the Cereoanhydride Structure Problem—Carboxylate Complications
Source: Mar Drugs. 2017 Jun 12;15(6):171. doi: 10.3390/md15060171 (PMC5484121; doi:10.3390/md15060171)
Supplement: Supplementary file 1 [file marinedrugs-15-00171-s001.pdf]

Supporting Information for:

Application of Computational Chemical Shift Prediction Techniques to the Cereoanhydride Structure Problem

C. M. Saunders and D. J. Tantillo*

University of California – Davis, 1 Shields Ave, Davis, CA, 95616, USA

djtantillo@ucdavis.edu

Table of Contents:

Energy profile for conformers of **1**: page 2

Computed chemical shifts for **1**: page 2

Energy profile for conformers of **4**: page 4

Computed chemical shifts for **4**: page 4

Energy profile for conformers of **2**: page 5

Computed chemical shifts for **2**: page 6

Energy profile for conformers of deprotonated **2**: page 7

Computed chemical shifts for deprotonated **2**: page 7

Computed chemical shifts for H bond models and dimer: page 8

Computed chemical shifts for acetic acid systems: page 16

Energy Comparison for B3LYP – D3 Optimizations: page 17

NMR Shift Table for B3LYP – D3 Optomized Geometries: page 17

Energy profile for conformers of **1** (B3LYP – D3): page 18

Computed chemical shifts for **1** (B3LYP – D3 geometry): page 18

Energy profile for conformers of **4** (B3LYP – D3): page 20

Computed chemical shifts for **4** (B3LYP – D3 geometry): page 20

Energy profile for conformers of **2** (B3LYP – D3): page 21

Computed chemical shifts for **2** (B3LYP – D3 geometry): page 22

Energy profile for conformers of deprotonated **2** (B3LYP – D3): page 23

Computed chemical shifts for deprotonated **2** (B3LYP – D3 geometry): page 23

Full Gaussian citations: page 24

Compound **1** (Anhydride):

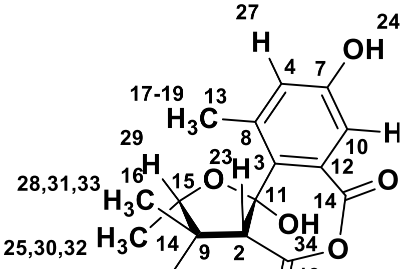

Energy profiles for the conformers found for compound **1**. The percent population is shown, with those included in the calculation highlighted in green. Data is sorted by relative Gibb's free energy.

B3LYP/6-31G(d)		
	ΔG (kcal/mol)	% population
4.log	0.000	52.2
5.log	0.576	19.7
1.log	0.701	16.0
2.log	1.164	7.3
3.log	1.409	4.8
8.log	46.995	

Computed chemical shifts (ppm) for compound **1**. Values were obtained by scaling the calculated isotropic values of individual atoms, using the scaling factors noted in the method section. The chemical shifts shown are Boltzmann weighted average of the corresponding conformers. Mean absolute deviations (MADs) (between the computed and experimental numbers, ppm) maximum absolute deviations (MAXs) are also included.

^{13}C			Isolation[1]		Synthesis[2]	
Expt. #s	Comp #	Comp.	Expt.	Abs. Dev.	Expt.	Abs. Dev.
C17	C9	47.61	43.8	3.8	43.8	3.8
C1	C2	65.73	60.7	5.0	60.4	5.3
C15	C15	85.57	87.5	1.9	87.4	1.8
C18	C16	24.82	26.2	1.4	26.1	1.3
C19	C1	16.01	17.3	1.3	17.2	1.2
C13	C3	133.89	137.5	3.6	137.4	3.5
C11	C8	139.77	136.1	3.7	136.2	3.6
C4	C12	132.73	131.0	1.7	131.0	1.7
C10	C4	123.54	124.6	1.1	124.7	1.2
C8	C10	115.88	108.6	7.3	108.6	7.3
C9	C7	156.62	160.9	4.3	161.0	4.4
C14	C11	103.51	113.3	9.8	113.1	9.6

C2	C5	168.94	170.4	1.5	170.4	1.5
C3	C6	168.72	170.8	2.1	170.7	2.0
C12	C13	22.62	17.6	5.0	17.6	5.0
C16	C14	14.55	15.0	0.4	14.9	0.3
			**MAD:**	**3.4**	**MAD:**	**3.3**

^{1}H			Isolation[1]		Synthesis[2]	
**Expt. #s**	**Comp #**	**Comp.**	**Expt.**	**Abs. Dev.**	**Expt.**	**Abs. Dev.**
C1	H23	3.58	3.55	0.03	3.51	0.07
C15	H29	4.17	4.24	0.07	4.21	0.04
C10	H27	7.11	6.97	0.14	6.95	0.16
C8	H26	7.10	6.99	0.11	6.93	0.17
-	H24		n.o.		n.o.	
-	H34		n.o.		n.o.	
C16	ave(25,30,32)	1.31	1.32	0.01	1.28	0.03
C18	ave(28,31,33)	1.31	1.38	0.07	1.36	0.05
C19	ave(20-22)	1.37	1.32	0.05	1.28	0.09
C12	ave(17-19)	2.54	2.24	0.30	2.38	0.16
			**MAD:**	**0.10**	**MAD:**	**0.10**

*Literature reference for experimental data.

Compound **4** (Intermediate):

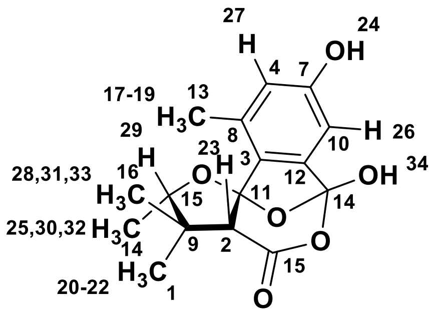

Energy profiles for the conformers found for compound **4**. The percent population is shown, with those included in the calculation highlighted in green. Data is sorted by relative Gibb's free energy.

B3LYP/6-31G(d)		
	ΔG (kcal/mol)	% population
1.log	0.000	60.5
2.log	0.284	37.5
4.log	2.414	0.9
3.log	2.484	1.0

8.log	127.76	

Computed chemical shifts (ppm) for compound **4**. Values were obtained by scaling the calculated isotropic values of individual atoms, using the scaling factors noted in the method section. The chemical shifts shown are Boltzmann weighted average of the corresponding conformers. Mean absolute deviations (MADs) (between the computed and experimental numbers, ppm) maximum absolute deviations (MAXs) are also included.

^{13}C			Isolation[1]		Synthesis[2]	
**Expt. #s**	**Comp #**	**Comp.**	**Expt.**	**Abs. Dev.**	**Expt.**	**Abs. Dev.**
C17	C9	47.67	43.8	3.9	43.8	3.9
C1	C2	61.87	60.7	1.2	60.4	1.5
C15	C15	88.07	87.5	0.6	87.4	0.7
C18	C16	24.64	26.2	1.6	26.1	1.5
C19	C1	16.70	17.3	0.6	17.2	0.5
C13	C3	131.18	137.5	6.3	137.4	6.2
C11	C8	137.48	136.1	1.4	136.2	1.3
C4	C12	140.46	131.0	9.5	131.0	9.5
C10	C4	118.33	124.6	6.3	124.7	6.4
C8	C10	104.43	108.6	4.2	108.6	4.2
C9	C7	158.21	160.9	2.7	161.0	2.8
C14	C11	112.38	113.3	0.9	113.1	0.7
C2	C5	17.75	17.6	0.1	17.6	0.1
C3	C6	116.66	170.8	54.1	170.7	54.0
C12	C13	173.61	170.4	3.2	170.4	3.2
C16	C14	13.99	15.0	1.0	14.9	0.9
			**MAD:**	**6.1**	**MAD:**	**6.1**

^{1}H			Isolation[1]		Synthesis[2]	
**Expt. #s**	**Comp #**	**Comp.**	**Expt.**	**Abs. Dev.**	**Expt.**	**Abs. Dev.**
C1	H23	3.27	3.55	0.28	3.51	0.24
C15	H29	4.46	4.24	0.22	4.21	0.25
C10	H27	6.84	6.97	0.13	6.95	0.11
C8	H26	6.93	6.99	0.06	6.93	0.00
-	H24	5.26	n.o.		n.o.	
-	H34	4.06	n.o.		n.o.	
C16	ave(25,30,32)	1.33	1.32	0.01	1.28	0.05
C18	ave(28,31,33)	1.18	1.38	0.20	1.36	0.18
C19	ave(20-22)	1.35	1.32	0.03	1.28	0.07
C12	ave(17-19)	2.398609	2.24	0.16	2.38	0.02
			**MAD:**	**0.14**	**MAD:**	**0.11**

*Literature reference for experimental data.

Compound **2** (Carboxylic Acid):

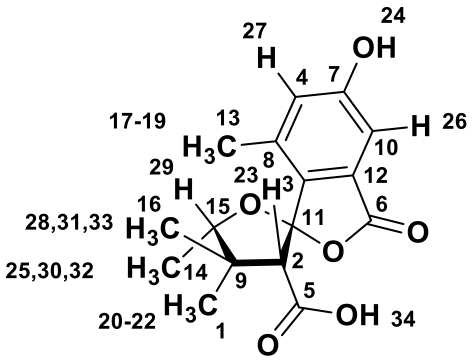

Energy profiles for the conformers found for compound **2**. The percent population is shown, with those included in the calculation highlighted in green. Data is sorted by relative Gibb's free energy.

B3LYP/6-31G(d)		
	ΔG (kcal/mol)	% population
2.log	0.000	51.3
1.log	0.491	22.4
3.log	0.626	17.8
4.log	1.295	5.8
9.log	1.752	2.7
10.log	2.331	1.0
5.log	4.357	
14.log	4.890	
6.log	4.948	
16.log	5.636	
13.log	7.155	
15.log	7.919	

Computed chemical shifts (ppm) for compound **2**. Values were obtained by scaling the calculated isotropic values of individual atoms, using the scaling factors noted in the method section. The chemical shifts shown are Boltzmann weighted average of the corresponding conformers. Mean absolute deviations (MADs) (between the computed and experimental numbers, ppm) maximum absolute deviations (MAXs) are also included.

^{13}C			Isolation[1]		Synthesis[2]	
**Expt. #s**	**Comp #**	**Comp.**	**Expt.**	**Abs. Dev.**	**Expt.**	**Abs. Dev.**
C17	C9	16.05	17.3	1.2	17.2	1.1
C1	C2	58.95	60.7	1.7	60.4	1.4

C15	C15	138.24	137.5	0.7	137.4	0.8
C18	C16	124.61	124.6	0.0	124.7	0.1
C19	C1	172.54	170.4	2.1	170.4	2.1
C13	C3	171.40	170.8	0.6	170.7	0.7
C11	C8	159.09	160.9	1.8	161.0	1.9
C4	C12	139.35	136.1	3.3	136.2	3.2
C10	C4	47.60	43.8	3.8	43.8	3.8
C8	C10	108.08	108.6	0.5	108.6	0.5
C9	C7	112.04	113.3	1.3	113.1	1.1
C14	C11	129.85	131.0	1.2	131.0	1.2
C2	C5	17.85	17.6	0.3	17.6	0.3
C3	C6	13.99	15.0	1.0	14.9	0.9
C12	C13	86.59	87.5	0.9	87.4	0.8
C16	C14	24.44	26.2	1.8	26.1	1.7
			**MAD:**	**1.4**	**MAD:**	**1.3**

*Literature reference for experimental data.

^{1}H			Isolation[1]		Synthesis[2]	
**Expt. #s**	**Comp #**	**Comp.**	**Expt.**	**Abs. Dev.**	**Expt.**	**Abs. Dev.**
C1	H23	4.05	3.55	0.50	3.51	0.54
C15	H29	4.38	4.24	0.14	4.21	0.17
C10	H27	7.24	6.97	0.27	6.95	0.29
C8	H26	7.12	6.99	0.13	6.93	0.19
-	H24	5.42	n.o.		n.o.	
-	H34	6.54	n.o.		n.o.	
C16	ave(25,30,32)	1.31	1.32	0.01	1.28	0.03
C18	ave(28,31,33)	1.38	1.38	0.00	1.36	0.02
C19	ave(20-22)	1.40	1.32	0.08	1.28	0.12
C12	ave(17-19)	2.64	2.24	0.40	2.38	0.26
			**MAD:**	**0.19**	**MAD:**	**0.20**

*Literature reference for experimental data.

Deprotonated Compound **2** (Carboxylate):

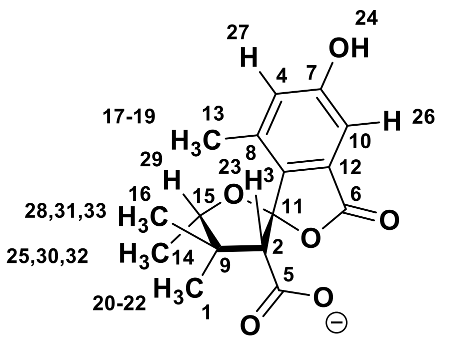

Energy profiles for the conformers found for deprotonated **2**. The percent population is shown, with those included in the calculation highlighted in green. Data is sorted by relative Gibb's free energy.

B3LYP/6-31G(d)		
	ΔG (kcal/mol)	% population
2.log	0.000	85.2
1.log	1.037	14.8

Computed chemical shifts (ppm) for deprotonated **2**. Values were obtained by scaling the calculated isotropic values of individual atoms, using the scaling factors noted in the method section. The chemical shifts shown are Boltzmann weighted average of the corresponding conformers. Mean absolute deviations (MADs) (between the computed and experimental numbers, ppm) maximum absolute deviations (MAXs) are also included.

^{13}C			Isolation[1]		Synthesis[2]	
**Expt. #s**	**Comp #**	**Comp.**	**Expt.**	**Abs. Dev.**	**Expt.**	**Abs. Dev.**
C17	C9	16.71	17.3	0.6	17.2	0.5
C1	C2	61.64	60.7	0.9	60.4	1.2
C15	C15	140.65	137.5	3.2	137.4	3.3
C18	C16	123.26	124.6	1.3	124.7	1.4
C19	C1	172.36	170.4	2.0	170.4	2.0
C13	C3	172.24	170.8	1.4	170.7	1.5
C11	C8	158.39	160.9	2.5	161.0	2.6
C4	C12	138.16	136.1	2.1	136.2	2.0
C10	C4	46.86	43.8	3.1	43.8	3.1
C8	C10	107.60	108.6	1.0	108.6	1.0
C9	C7	116.64	113.3	3.3	113.1	3.5
C14	C11	131.16	131.0	0.2	131.0	0.2
C2	C5	17.83	17.6	0.2	17.6	0.2
C3	C6	15.14	15.0	0.1	14.9	0.2
C12	C13	85.85	87.5	1.6	87.4	1.5
C16	C14	26.02	26.2	0.2	26.1	0.1
			**MAD:**	**1.5**	**MAD:**	**1.5**

*Literature reference for experimental data.

^{1}H			Isolation[1]		Synthesis[2]	
**Expt. #s**	**Comp #**	**Comp.**	**Expt.**	**Abs. Dev.**	**Expt.**	**Abs. Dev.**
C1	H23	3.66	3.55	0.11	3.51	0.15
C15	H29	4.23	4.24	0.01	4.21	0.02
C10	H27	7.18	6.97	0.21	6.95	0.23

C8	H26	7.05	6.99	0.06	6.93	0.12
-	H24	5.23	n.o.		n.o.	
C16	H34	1.29	1.32	0.03	1.28	0.01
C18	ave(25,30,32)	1.47	1.38	0.09	1.36	0.11
C19	ave(28,31,33)	1.42	1.32	0.10	1.28	0.14
C12	ave(20-22)	2.49	2.24	0.25	2.38	0.11
	ave(17-19)		**MAD:**	**0.11**	**MAD:**	**0.11**

*Literature reference for experimental data.

Computed chemical shifts for H-Bonding models:

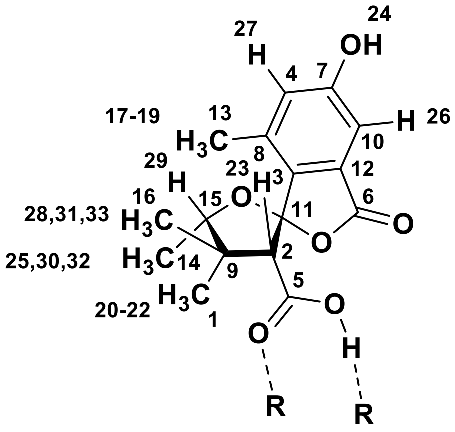

H-bonds to water:

Energy profiles for the conformers found for water H-bonding model. The percent population is shown, with those included in the calculation highlighted in green. Data is sorted by relative Gibb's free energy.

B3LYP/6-31G(d)		
	ΔG (kcal/mol)	% population
water_up.log	0	85.7
water_down.log	1.06173	14.3

Computed chemical shifts (ppm) for water H-bonding model. Values were obtained by scaling the calculated isotropic values of individual atoms, using the scaling factors noted in the method section. The chemical shifts shown are Boltzmann weighted average of the corresponding conformers. Mean absolute deviations (MADs) (between the computed and experimental numbers, ppm) maximum absolute deviations (MAXs) are also included.

Water up

^{13}C			Isolation[1]		Synthesis[2]	
**Expt. #s**	**Comp #**	**Comp.**	**Expt.**	**Abs. Dev.**	**Expt.**	**Abs. Dev.**
C17	C9	15.25	17.3	2.1	17.2	2.0

C1	C2	59.46	60.7	1.2	60.4	0.9
C15	C15	138.99	137.5	1.5	137.4	1.6
C18	C16	124.68	124.6	0.1	124.7	0.0
C19	C1	175.90	170.4	5.5	170.4	5.5
C13	C3	171.30	170.8	0.5	170.7	0.6
C11	C8	159.19	160.9	1.7	161.0	1.8
C4	C12	139.11	136.1	3.0	136.2	2.9
C10	C4	47.09	43.8	3.3	43.8	3.3
C8	C10	107.72	108.6	0.9	108.6	0.9
C9	C7	112.51	113.3	0.8	113.1	0.6
C14	C11	129.36	131.0	1.6	131.0	1.6
C2	C5	17.64	17.6	0.0	17.6	0.0
C3	C6	13.82	15.0	1.2	14.9	1.1
C12	C13	86.90	87.5	0.6	87.4	0.5
C16	C14	23.99	26.2	2.2	26.1	2.1
			**MAD:**	**1.6**	**MAD:**	**1.6**

*Literature reference for experimental data.

^{1}H			Isolation[1]		Synthesis[2]	
**Expt. #s**	**Comp #**	**Comp.**	**Expt.**	**Abs. Dev.**	**Expt.**	**Abs. Dev.**
C1	H23	4.06	3.55	0.5	3.51	0.5
C15	H29	4.32	4.24	0.1	4.21	0.1
C10	H27	7.24	6.97	0.3	6.95	0.3
C8	H26	7.09	6.99	0.1	6.93	0.2
-	H24	5.39	n.o.		n.o.	
-	H34	10.53	n.o.		n.o.	
C16	ave(25,30,32)	1.28	1.32	0.0	1.28	0.0
C18	ave(28,31,33)	1.36	1.38	0.0	1.36	0.0
C19	ave(20-22)	1.42	1.32	0.1	1.28	0.1
C12	ave(17-19)	2.51	2.42	0.1	2.38	0.1
			**MAD:**	**0.15**	**MAD:**	**0.17**

*Literature reference for experimental data.

Water down

^{13}C			Isolation[1]		Synthesis[2]	
**Expt. #s**	**Comp #**	**Comp.**	**Expt.**	**Abs. Dev.**	**Expt.**	**Abs. Dev.**
C17	C9	15.02	17.3	2.3	17.2	2.2
C1	C2	59.54	60.7	1.2	60.4	0.9
C15	C15	139.28	137.5	1.8	137.4	1.9
C18	C16	124.69	124.6	0.1	124.7	0.0

C19	C1	173.01	170.4	2.6	170.4	2.6
C13	C3	173.38	170.8	2.6	170.7	2.7
C11	C8	158.94	160.9	2.0	161.0	2.1
C4	C12	139.15	136.1	3.1	136.2	3.0
C10	C4	46.07	43.8	2.3	43.8	2.3
C8	C10	107.45	108.6	1.2	108.6	1.2
C9	C7	114.41	113.3	1.1	113.1	1.3
C14	C11	130.49	131.0	0.5	131.0	0.5
C2	C5	18.21	17.6	0.6	17.6	0.6
C3	C6	13.57	15.0	1.4	14.9	1.3
C12	C13	86.96	87.5	0.5	87.4	0.4
C16	C14	22.36	26.2	3.8	26.1	3.7
			**MAD:**	**1.7**	**MAD:**	**1.7**

*Literature reference for experimental data.

^{1}H			Isolation[1]		Synthesis[2]	
**Expt. #s**	**Comp #**	**Comp.**	**Expt.**	**Abs. Dev.**	**Expt.**	**Abs. Dev.**
C1	H23	3.93	3.55	0.4	3.51	0.4
C15	H29	4.37	4.24	0.1	4.21	0.2
C10	H27	7.26	6.97	0.3	6.95	0.3
C8	H26	7.12	6.99	0.1	6.93	0.2
-	H24	5.43	n.o.		n.o.	
-	H34	9.43	n.o.		n.o.	
C16	ave(25,30,32)	1.29	1.32	0.0	1.28	0.0
C18	ave(28,31,33)	1.29	1.38	0.1	1.36	0.1
C19	ave(20-22)	1.38	1.32	0.1	1.28	0.1
C12	ave(17-19)	2.50	2.42	0.1	2.38	0.1
			**MAD:**	**0.15**	**MAD:**	**0.17**

*Literature reference for experimental data.

H-bonds to methanol:

Energy profiles for the conformers found for methanol H-bonding model. The percent population is shown, with those included in the calculation highlighted in green. Data is sorted by relative Gibb's free energy.

B3LYP/6-31G(d)		
	ΔG (kcal/mol)	% population
methanol_up.log	0	95.1
methanol_down.log	1.763275	4.9

Computed chemical shifts (ppm) for compound methanol H-bonding model. Values were obtained by scaling the calculated isotropic values of individual atoms, using the scaling factors noted in the method section. The chemical shifts shown are Boltzmann weighted average of the corresponding conformers. Mean absolute deviations (MADs) (between the computed and experimental numbers, ppm) maximum absolute deviations (MAXs) are also included.

MeOH up

^{13}C			Isolation[1]		Synthesis[2]	
**Expt. #s**	**Comp #**	**Comp.**	**Expt.**	**Abs. Dev.**	**Expt.**	**Abs. Dev.**
C17	C9	10.60	17.3	6.7	17.2	6.6
C1	C2	53.14	60.7	7.6	60.4	7.3
C15	C15	126.51	137.5	11.0	137.4	10.9
C18	C16	109.70	124.6	14.9	124.7	15.0
C19	C1	159.45	170.4	10.9	170.4	10.9
C13	C3	154.76	170.8	16.0	170.7	15.9
C11	C8	143.72	160.9	17.2	161.0	17.3
C4	C12	125.64	136.1	10.5	136.2	10.6
C10	C4	40.76	43.8	3.0	43.8	3.0
C8	C10	93.96	108.6	14.6	108.6	14.6
C9	C7	103.63	113.3	9.7	113.1	9.5
C14	C11	116.64	131.0	14.4	131.0	14.4
C2	C5	12.41	17.6	5.2	17.6	5.2
C3	C6	9.32	15.0	5.7	14.9	5.6
C12	C13	78.40	87.5	9.1	87.4	9.0
C16	C14	18.38	26.2	7.8	26.1	7.7
			**MAD:**	**10.3**	**MAD:**	**10.2**

*Literature reference for experimental data.

^{1}H			Isolation[1]		Synthesis[2]	
**Expt. #s**	**Comp #**	**Comp.**	**Expt.**	**Abs. Dev.**	**Expt.**	**Abs. Dev.**
C1	H23	4.02	3.55	0.5	3.51	0.5
C15	H29	4.31	4.24	0.1	4.21	0.1
C10	H27	7.09	6.97	0.1	6.95	0.1
C8	H26	7.04	6.99	0.0	6.93	0.1
-	H24	5.07	n.o.		n.o.	
-	H34	10.90	n.o.		n.o.	
C16	ave(25,30,32)	1.32	1.32	0.0	1.28	0.0
C18	ave(28,31,33)	1.39	1.38	0.0	1.36	0.0
C19	ave(20-22)	1.46	1.32	0.1	1.28	0.2
C12	ave(17-19)	2.53	2.42	0.1	2.38	0.1
			**MAD:**	**0.12**	**MAD:**	**0.16**

MeOH down

^{13}C			Isolation[1]		Synthesis[2]	
**Expt. #s**	**Comp #**	**Comp.**	**Expt.**	**Abs. Dev.**	**Expt.**	**Abs. Dev.**
C17	C9	10.18	17.3	7.1	17.2	7.0
C1	C2	53.05	60.7	7.7	60.4	7.4
C15	C15	126.58	137.5	10.9	137.4	10.8
C18	C16	109.98	124.6	14.6	124.7	14.7
C19	C1	155.64	170.4	14.8	170.4	14.8
C13	C3	156.83	170.8	14.0	170.7	13.9
C11	C8	143.75	160.9	17.1	161.0	17.2
C4	C12	125.40	136.1	10.7	136.2	10.8
C10	C4	40.88	43.8	2.9	43.8	2.9
C8	C10	93.48	108.6	15.1	108.6	15.1
C9	C7	105.24	113.3	8.1	113.1	7.9
C14	C11	118.03	131.0	13.0	131.0	13.0
C2	C5	12.72	17.6	4.9	17.6	4.9
C3	C6	9.00	15.0	6.0	14.9	5.9
C12	C13	79.41	87.5	8.1	87.4	8.0
C16	C14	17.41	26.2	8.8	26.1	8.7
			**MAD:**	**10.2**	**MAD:**	**10.2**

*Literature reference for experimental data.

^{1}H			Isolation[1]		Synthesis[2]	
**Expt. #s**	**Comp #**	**Comp.**	**Expt.**	**Abs. Dev.**	**Expt.**	**Abs. Dev.**
C1	H23	3.84	3.55	0.29	3.51	0.33
C15	H29	4.31	4.24	0.07	4.21	0.10
C10	H27	7.10	6.97	0.13	6.95	0.15
C8	H26	7.00	6.99	0.01	6.93	0.07
-	H24	5.09	n.o.		n.o.	
-	H34	9.59	n.o.		n.o.	
C16	ave(25,30,32)	1.38	1.32	0.06	1.28	0.10
C18	ave(28,31,33)	1.34	1.38	0.04	1.36	0.02
C19	ave(20-22)	1.47	1.32	0.15	1.28	0.19
C12	ave(17-19)	2.53	2.42	0.11	2.38	0.15
			**MAD:**	**0.11**	**MAD:**	**0.14**

*Literature reference for experimental data.

Dimer model:

Computed chemical shifts (ppm) for dimer model. Values were obtained by scaling the calculated isotropic values of individual atoms, using the scaling factors noted in the method section. The chemical shifts shown are Boltzmann weighted average of the corresponding conformers. Mean absolute deviations (MADs) (between the computed and experimental numbers, ppm) maximum absolute deviations (MAXs) are also included.

^{13}C			Isolation[1]		Synthesis[2]	
**Expt. #s**	**Comp #**	**Comp.**	**Expt.**	**Abs. Dev.**	**Expt.**	**Abs. Dev.**
C17	C9	15.21	17.3	2.1	17.2	2.0
C1	C2	59.42	60.7	1.3	60.4	1.0
C15	C15	138.77	137.5	1.3	137.4	1.4
C18	C16	124.83	124.6	0.2	124.7	0.1
C19	C1	178.27	170.4	7.9	170.4	7.9
C13	C3	171.48	170.8	0.7	170.7	0.8
C11	C8	159.30	160.9	1.6	161.0	1.7
C4	C12	139.42	136.1	3.3	136.2	3.2
C10	C4	46.41	43.8	2.6	43.8	2.6
C8	C10	107.94	108.6	0.7	108.6	0.7
C9	C7	112.03	113.3	1.3	113.1	1.1
C14	C11	128.55	131.0	2.4	131.0	2.4
C2	C5	17.83	17.6	0.2	17.6	0.2
C3	C6	13.65	15.0	1.4	14.9	1.3
C12	C13	86.77	87.5	0.7	87.4	0.6
C16	C14	23.55	26.2	2.7	26.1	2.6
			**MAD:**	**1.9**	**MAD:**	**1.8**

*Literature reference for experimental data.

^{1}H			Isolation[1]		Synthesis[2]	
**Expt. #s**	**Comp #**	**Comp.**	**Expt.**	**Abs. Dev.**	**Expt.**	**Abs. Dev.**
C1	H23	4.11	3.55	0.6	3.51	0.6
C15	H29	4.38	4.24	0.1	4.21	0.2
C10	H27	7.17	6.97	0.2	6.95	0.2
C8	H26	7.01	6.99	0.0	6.93	0.1
-	H24	5.32	n.o.		n.o.	
-	H34	12.41	n.o.		n.o.	
C16	ave(25,30,32)	1.28	1.32	0.0	1.28	0.0
C18	ave(28,31,33)	1.36	1.38	0.0	1.36	0.0
C19	ave(20-22)	1.41	1.32	0.1	1.28	0.1
C12	ave(17-19)	2.48	2.42	0.1	2.38	0.1
			**MAD:**	**0.14**	**MAD:**	**0.16**

Dimer:

Computed chemical shifts (ppm) for dimer. Values were obtained by scaling the calculated isotropic values of individual atoms, using the scaling factors noted in the method section. The chemical shifts shown are Boltzmann weighted average of the corresponding conformers. Mean absolute deviations (MADs) (between the computed and experimental numbers, ppm) maximum absolute deviations (MAXs) are also included.

^{13}C			Isolation[1]		Synthesis[2]	
**Expt. #s**	**Comp #**	**Comp.**	**Expt.**	**Abs. Dev.**	**Expt.**	**Abs. Dev.**
C17	C9	15.81	17.3	1.5	17.2	1.4
C1	C2	59.43	60.7	1.3	60.4	1.0
C15	C15	138.68	137.5	1.2	137.4	1.3
C18	C16	124.88	124.6	0.3	124.7	0.2
C19	C1	178.42	170.4	8.0	170.4	8.0
C13	C3	171.36	170.8	0.6	170.7	0.7
C11	C8	159.08	160.9	1.8	161.0	1.9
C4	C12	139.09	136.1	3.0	136.2	2.9
C10	C4	46.85	43.8	3.1	43.8	3.1
C8	C10	108.10	108.6	0.5	108.6	0.5
C9	C7	111.74	113.3	1.6	113.1	1.4
C14	C11	128.98	131.0	2.0	131.0	2.0
C2	C5	17.49	17.6	0.1	17.6	0.1
C3	C6	14.08	15.0	0.9	14.9	0.8
C12	C13	86.80	87.5	0.7	87.4	0.6
C16	C14	23.89	26.2	2.3	26.1	2.2
			**MAD:**	**1.8**	**MAD:**	**1.7**

*Literature reference for experimental data.

^{1}H			Isolation[1]		Synthesis[2]	
**Expt. #s**	**Comp #**	**Comp.**	**Expt.**	**Abs. Dev.**	**Expt.**	**Abs. Dev.**
C1	H23	4.02	3.55	0.47	3.51	0.51
C15	H29	4.30	4.24	0.1	4.21	0.1
C10	H27	7.26	6.97	0.3	6.95	0.3
C8	H26	7.05	6.99	0.1	6.93	0.1
-	H24	5.35	n.o.		n.o.	
-	H34	12.49	n.o.		n.o.	
C16	ave(25,30,32)	1.24	1.32	0.1	1.28	0.0
C18	ave(28,31,33)	1.30	1.38	0.1	1.36	0.1
C19	ave(20-22)	1.36	1.32	0.0	1.28	0.1
C12	ave(17-19)	2.49	2.42	0.1	2.38	0.1
			**MAD:**	**0.14**	**MAD:**	**0.17**

*Literature reference for experimental data.

Carboxylate-acid complex:

Computed chemical shifts (ppm) for carboxylate-acid complex. Values were obtained by scaling the calculated isotropic values of individual atoms, using the scaling factors noted in the method section. The chemical shifts shown are Boltzmann weighted average of the corresponding conformers. Mean absolute deviations (MADs) (between the computed and experimental numbers, ppm) maximum absolute deviations (MAXs) are also included.

^{13}C			Isolation[1]		Synthesis[2]	
**Expt. #s**	**Comp #**	**Comp.**	**Expt.**	**Abs. Dev.**	**Expt.**	**Abs. Dev.**
19	C17	21.25	17.3	4.0	17.2	4.1
1	C1	60.31	60.7	0.4	60.4	0.1
13	C15	139.16	137.5	1.7	137.4	1.8
10	C18	123.30	124.6	1.3	124.7	1.4
2	C19	174.15	170.4	3.7	170.4	3.7
3	C13	171.95	170.8	1.2	170.7	1.3
9	C11	158.00	160.9	2.9	161.0	3.0
11	C4	139.76	136.1	3.7	136.2	3.6
17	C10	46.27	43.8	2.5	43.8	2.5
8	C8	107.84	108.6	0.8	108.6	0.8
14	C9	114.72	113.3	1.4	113.1	1.6
4	C14	130.33	131.0	0.7	131.0	0.7
12	C2	17.71	17.6	0.1	17.6	0.1
16	C3	15.44	15.0	0.4	14.9	0.5
15	C12	86.59	87.5	0.9	87.4	0.8
18	C16	21.09	26.2	5.1	26.1	5.0
			**MAD:**	**1.9**	**MAD:**	**1.9**

*Literature reference for experimental data.

^{1}H			Isolation[1]		Synthesis[2]	
**Expt. #s**	**Comp #**	**Comp.**	**Expt.**	**Abs. Dev.**	**Expt.**	**Abs. Dev.**
C1	H23	3.80	3.55	0.25	3.51	0.29
C15	H29	4.31	4.24	0.07	4.21	0.10
C10	H27	7.10	6.97	0.13	6.95	0.15
C8	H26	7.02	6.99	0.03	6.93	0.09
-	H24	5.49	n.o.		n.o.	
-	H34	11.21	n.o.		n.o.	
C16	ave(25,30,32)	1.23	1.32	0.09	1.28	0.05
C18	ave(28,31,33)	1.37	1.38	0.01	1.36	0.01
C19	ave(20-22)	1.44	1.32	0.12	1.28	0.16
C12	ave(17-19)	2.46	2.42	0.04	2.38	0.08
			**MAD:**	**0.09**	**MAD:**	**0.12**

*Literature reference for experimental data.

<u>Computed Chemical Shifts (ppm) for Acetic Acid-Based Systems</u>:

Values were obtained by scaling the calculated isotropic values of individual atoms, using the scaling factors noted in the method section. Deviations are explored for the carbonyl carbon. The chemical shifts shown for diacetate are Boltzmann weighted average of the corresponding conformers.

		Isotropic	Shift	Averaged	Reported[3-4]	Difference
Acetate	**1C**	**0.2520**	**180.6**		**182.6**	**2.0**
	**2C**	**158.6328**	**26.4**		**24.9**	**-1.5**
	3O	5.1169	-			
	4O	21.1203	-			
	5H	29.5719	2.14	1.98	1.80	-0.18
	6H	29.5720	2.14			
	7H	30.1019	1.65			

		Isotropic	Shift	Averaged	Reported[3, 5]	Difference
Acetic Acid	**1C**	**1.9432**	**178.9**		**178.1**	**-0.8**
	**2C**	**165.7104**	**19.5**		**22.0**	**2.5**
	3O	-61.2251	-			
	4O	115.2616	-			
	5H	24.7770	6.6			
	6H	29.2433	2.45	2.25	2.08	-0.17
	7H	29.2433	2.45			
	8H	29.8746	1.86			

Biacetate	Isotropic	Isotropic	Shift	Shift	Averaged	Reported[6]	Difference
	Conf_1	Conf_2	Conf_1	Conf_2			
E(kcal/mol)	0	2.0048625					
**1 C**	**-0.1413**	**-0.3938**	**180.9**	**181.2**	**181.3**	**180.2**	**-1.1**
**2 C**	**161.0401**	**159.888**	**24.1**	**25.2**	**24.4**		
**3 C**	**-0.8501**	**-0.8136**	**181.6**	**181.6**			
**4 C**	**160.4052**	**159.7281**	**24.7**	**25.4**			
5 O	-28.6268	-5.8805					
6 O	61.8756	20.0211					
7 O	-18.0544	-44.2923					
8 O	41.5098	80.2739					
9 H	11.4201	14.5844					

10 H	29.4106	29.5627					
11 H	29.4782	29.9739					
12 H	30.0744	29.5627					
13 H	29.448	28.9227					
14 H	29.5238	28.9227					
15 H	30.1044	29.7839					

Computed Chemical Shifts (ppm) for Propanoic Acid-Based Systems:

Values were obtained by scaling the calculated isotropic values of individual atoms, using the scaling factors noted in the method section. The chemical shifts shown are Boltzmann weighted average of the corresponding conformers.

Propanoic Acid	Isotropic	Isotropic	Shift	Shift	Averaged	Reported[3, 7]	Difference
	Conf_1	Conf_2					
E (kcal/mol)	0.00	0.90					
**1C**	**-1.1**	**-1.3**	**181.9**	**182.0**	**181.9**	**181.3**	**-0.6**
**2C**	**157.4**	**154.4**	**27.6**	**30.6**	**28.1**	**28.7**	**0.6**
**3C**	**178.6**	**174.4**	**7.0**	**11.0**	**7.7**	**9.9**	**2.2**
4O	-52.2	-57.3	-	-	-		
5O	119.6	118.0	-	-	-		
6H	24.7	24.8	6.68	6.65			
7H	28.9	29.5	2.73	2.24	2.68	2.38	-0.30
8H	28.9	29.0	2.73	2.71			
9H	30.7	30.4	1.09	1.38	1.15	1.16	0.01
10H	30.7	30.3	1.11	1.44			
11H	30.7	30.5	1.11	1.28			

		**Isotropic**	**Shift**	**Averaged**	**Reported[3, 8]**	**Difference**
Propanoate	**1C**	**-2.1**	**182.8**		**186.0**	**3.2**
	**2C**	**151.5**	**33.4**		**32.2**	**-1.2**
	**3C**	**175.0**	**10.5**		**11.7**	**1.2**
	4O	19.9	-			
	5O	30.8	-			
	6H	29.4	2.27	2.27	2.18	-0.09
	7H	29.4	2.27			
	8H	30.7	1.09	1.04	1.06	0.02
	9H	30.8	1.02			

	10H	30.8	1.02			

Energy Comparison for B3LYP – D3 Optimizations:

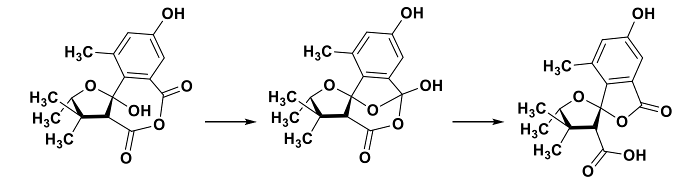

proposed cereoanhydride (**1**) 0 kcal/mol — **4** -1.55 kcal/mol — cereoanhydride (**2**) -21.9 kcal/mol

NMR Shift Table for B3LYP – D3 Optimizations:

Atom Label	Exp. δ	Anhydride (1) δ	Abs. Dev.	Acid (2) δ	Abs. Dev.	Carboxylate δ	Abs. Dev.
C6	170.7	168.8	1.9	168.6	2.1	172.2	1.5
C5	170.4	168.7	1.7	170.1	0.3	172.6	2.2
C7	161.0	156.8	4.2	156.8	4.2	158.4	2.6
C3	137.4	133.8	3.6	135.7	1.7	140.3	2.9
C8	136.2	140.0	3.8	136.8	0.6	137.5	1.3
C12	131.0	132.2	1.2	127.4	3.6	131.6	0.6
C4	124.7	123.4	1.3	122.5	2.2	122.7	2.0
C11	113.1	103.2	***9.9***	109.9	3.2	115.6	2.5
C10	108.6	115.6	***7.0***	106.5	2.1	107.6	1.0
C15	87.4	85.0	2.4	85.2	2.2	85.5	1.9
C2	60.4	65.4	5.0	58.4	2.0	60.9	0.5
C9	43.8	46.7	2.9	46.8	3.0	46.5	2.7
C16	26.1	24.0	2.1	23.8	2.3	25.6	0.5
C13	17.6	22.4	4.8	17.1	0.5	17.3	0.3
C1	17.2	15.6	1.6	16.2	1.0	16.6	0.6
C14	14.9	14.1	0.8	14.0	0.9	14.9	0.0
	M.A.D. [2]		3.4		2.0		1.4
	MAX [3]		***9.9***		4.2		2.9
H27	6.95	7.11	0.16	6.95	0.14	7.11	0.16
H26	6.93	7.09	0.16	6.93	0.06	7.09	0.16
H29	4.21	4.09	0.12	4.21	0.13	4.24	0.03
H23	3.51	3.53	0.02	3.51	0.***42***	3.54	0.03
H17-19	2.28	2.56	0.18	2.38	0.09	2.48	0.10
H28,31,33	1.36	1.29	0.07	1.36	0.01	1.46	0.10
H30,32,25	1.28	1.30	0.02	1.28	0.02	1.32	0.04
H20-22	1.28	1.36	0.08	1.28	0.07	1.39	0.11
	M.A.D. [2]		0.10		0.12		0.09
	MAX [3]		0.18		***0.42***		0.16

[1] Protons not seen in the experimental NMRs are not included here (OH's and acid H). [2] Mean absolute deviation. [3] Maximum absolute deviation. Deviations of <5 ppm (^{13}C) and <0.3 ppm (^{1}H) are generally considered acceptable.[9-13]

Energy profile and computed chemical shifts for B3LYP-D3 optimized structures:

Compound **1** (Anhydride):

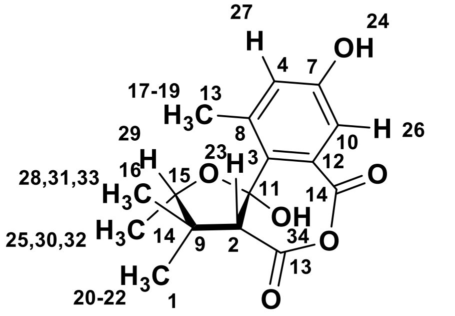

Energy profiles for the conformers found for compound **1**. The percent population is shown, with those included in the calculation highlighted in green. Data is sorted by relative Gibb's free energy.

B3LYP/6-31G(d)		
	ΔG (kcal/mol)	% population
8.log	0.000	48.9
5.log	0.511	20.6
1.log	0.556	19.1
2.log	1.154	7.0
3.log	1.443	4.3
9.log	216.628	

Computed chemical shifts (ppm) for compound **1**. Values were obtained by scaling the calculated isotropic values of individual atoms, using the scaling factors noted in the method section. The chemical shifts shown are Boltzmann weighted average of the corresponding conformers. Mean absolute deviations (MADs) (between the computed and experimental numbers, ppm) maximum absolute deviations (MAXs) are also included.

^{13}C			Isolation[1]		Synthesis[2]	
Expt. #s	Comp #	Comp.	Expt.	Abs. Dev.	Expt.	Abs. Dev.
C17	C9	46.70	43.8	2.9	43.8	2.9
C1	C2	65.44	60.7	4.7	60.4	5.0
C15	C15	84.97	87.5	2.5	87.4	2.4
C18	C16	23.97	26.2	2.2	26.1	2.1
C19	C1	15.59	17.3	1.7	17.2	1.6
C13	C3	133.84	137.5	3.7	137.4	3.6

C11	C8	140.01	136.1	3.9	136.2	3.8
C4	C12	132.24	131.0	1.2	131.0	1.2
C10	C4	123.39	124.6	1.2	124.7	1.3
C8	C10	115.64	108.6	7.0	108.6	7.0
C9	C7	156.77	160.9	4.1	161.0	4.2
C14	C11	103.20	113.3	10.1	113.1	9.9
C2	C5	168.66	170.4	1.7	170.4	1.7
C3	C6	168.79	170.8	2.0	170.7	1.9
C12	C13	22.36	17.6	4.8	17.6	4.8
C16	C14	14.07	15.0	0.9	14.9	0.8
			**MAD:**	**3.4**	**MAD:**	**3.4**

*Literature reference for experimental data.

^{1}H			Isolation[1]		Synthesis[2]	
**Expt. #s**	**Comp #**	**Comp.**	**Expt.**	**Abs. Dev.**	**Expt.**	**Abs. Dev.**
C1	H23	3.53	3.55	0.02	3.51	0.02
C15	H29	4.09	4.24	0.15	4.21	0.12
C10	H27	7.11	6.97	0.14	6.95	0.16
C8	H26	7.09	6.99	0.10	6.93	0.16
-	H24		n.o.		n.o.	
-	H34		n.o.		n.o.	
C16	ave(25,30,32)	1.30	1.32	0.02	1.28	0.02
C18	ave(28,31,33)	1.29	1.38	0.09	1.36	0.07
C19	ave(20-22)	1.36	1.32	0.04	1.28	0.08
C12	ave(17-19)	2.56	2.24	0.32	2.38	0.18
			**MAD:**	**0.11**	**MAD:**	**0.10**

*Literature reference for experimental data.

Compound **4** (Intermediate):

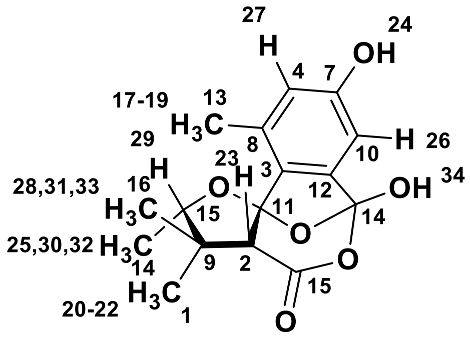

Energy profiles for the conformers found for compound **4**. The percent population is shown, with those included in the calculation highlighted in green. Data is sorted by relative Gibb's free energy.

B3LYP/6-31G(d)		
	ΔG (kcal/mol)	% population
1.log	0.000	62.7
2.log	0.353	34.6
5.log	2.112	1.8
4.log	2.450	1.0
3.log	2.686	

Computed chemical shifts (ppm) for compound **4**. Values were obtained by scaling the calculated isotropic values of individual atoms, using the scaling factors noted in the method section. The chemical shifts shown are Boltzmann weighted average of the corresponding conformers. Mean absolute deviations (MADs) (between the computed and experimental numbers, ppm) maximum absolute deviations (MAXs) are also included.

^{13}C			Isolation[1]		Synthesis[2]	
**Expt. #s**	**Comp #**	**Comp.**	**Expt.**	**Abs. Dev.**	**Expt.**	**Abs. Dev.**
C17	C9	46.73	43.8	2.9	43.8	2.9
C1	C2	61.83	60.7	1.1	60.4	1.4
C15	C15	87.52	87.5	0.0	87.4	0.1
C18	C16	23.93	26.2	2.3	26.1	2.2
C19	C1	16.46	17.3	0.8	17.2	0.7
C13	C3	131.36	137.5	6.1	137.4	6.0
C11	C8	137.13	136.1	1.0	136.2	0.9
C4	C12	139.89	131.0	8.9	131.0	8.9
C10	C4	117.97	124.6	6.6	124.7	6.7
C8	C10	104.03	108.6	4.6	108.6	4.6
C9	C7	158.49	160.9	2.4	161.0	2.5
C14	C11	111.74	113.3	1.6	113.1	1.4
C2	C5	17.51	17.6	0.1	17.6	0.1
C3	C6	117.59	170.8	53.2	170.7	53.1
C12	C13	173.59	170.4	3.2	170.4	3.2
C16	C14	13.86	15.0	1.1	14.9	1.0
			**MAD:**	**6.0**	**MAD:**	**6.0**

^{1}H			Isolation[1]		Synthesis[2]	
**Expt. #s**	**Comp #**	**Comp.**	**Expt.**	**Abs. Dev.**	**Expt.**	**Abs. Dev.**
C1	H23	3.23	3.55	0.32	3.51	0.28
C15	H29	4.44	4.24	0.20	4.21	0.23
C10	H27	6.84	6.97	0.13	6.95	0.11
C8	H26	6.93	6.99	0.06	6.93	0.00
-	H24	5.27	n.o.		n.o.	
-	H34	4.09	n.o.		n.o.	

C16	ave(25,30,32)	1.32	1.32	0.00	1.28	0.04
C18	ave(28,31,33)	1.17	1.38	0.21	1.36	0.19
C19	ave(20-22)	1.35	1.32	0.03	1.28	0.07
C12	ave(17-19)	2.42	2.24	0.18	2.38	0.04
			**MAD:**	**0.14**	**MAD:**	**0.12**

*Literature reference for experimental data.

Compound **2** (Carboxylic Acid):

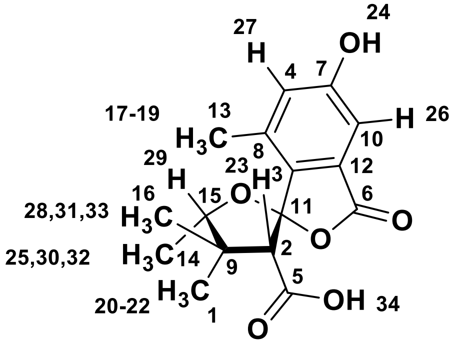

Energy profiles for the conformers found for compound **2**. The percent population is shown, with those included in the calculation highlighted in green. Data is sorted by relative Gibb's free energy.

B3LYP/6-31G(d)		
	ΔG (kcal/mol)	% population
2.log	0	44
1.log	0.284	27.3
3.log	0.635	15.1
4.log	1.035	7.7
9.log	1.381	4.3
10.log	1.999	1.5
8.log	4.14	
5.log	4.59	
6.log	5.22	
7.log	4.95	
12.log	6.86	
14.log	7.69	

Computed chemical shifts (ppm) for compound **2**. Values were obtained by scaling the calculated isotropic values of individual atoms, using the scaling factors noted in the method section. The chemical shifts shown are Boltzmann weighted average of the corresponding conformers. Mean absolute deviations (MADs) (between the computed and experimental numbers, ppm) maximum absolute deviations (MAXs) are also included.

^{13}C			Isolation[1]		Synthesis[2]	
**Expt. #s**	**Comp #**	**Comp.**	**Expt.**	**Abs. Dev.**	**Expt.**	**Abs. Dev.**
C17	C9	16.18	17.3	1.1	17.2	1.0
C1	C2	58.38	60.7	2.3	60.4	2.0
C15	C15	135.66	137.5	1.8	137.4	1.7
C18	C16	122.51	124.6	2.1	124.7	2.2
C19	C1	170.12	170.4	0.3	170.4	0.3
C13	C3	168.63	170.8	2.2	170.7	2.1
C11	C8	156.81	160.9	4.1	161.0	4.2
C4	C12	136.80	136.1	0.7	136.2	0.6
C10	C4	46.83	43.8	3.0	43.8	3.0
C8	C10	106.47	108.6	2.1	108.6	2.1
C9	C7	109.87	113.3	3.4	113.1	3.2
C14	C11	127.42	131.0	3.6	131.0	3.6
C2	C5	17.06	17.6	0.5	17.6	0.5
C3	C6	14.03	15.0	1.0	14.9	0.9
C12	C13	85.23	87.5	2.3	87.4	2.2
C16	C14	23.85	26.2	2.4	26.1	2.3
			**MAD:**	**2.1**	**MAD:**	**2.0**

*Literature reference for experimental data.

^{1}H			Isolation[1]		Synthesis[2]	
**Expt. #s**	**Comp #**	**Comp.**	**Expt.**	**Abs. Dev.**	**Expt.**	**Abs. Dev.**
C1	H23	3.93	3.55	0.38	3.51	0.42
C15	H29	4.34	4.24	0.10	4.21	0.13
C10	H27	7.09	6.97	0.12	6.95	0.14
C8	H26	6.99	6.99	0.00	6.93	0.06
-	H24	5.32	n.o.		n.o.	
-	H34	6.89	n.o.		n.o.	
C16	ave(25,30,32)	1.30	1.32	0.02	1.28	0.02
C18	ave(28,31,33)	1.37	1.38	0.01	1.36	0.01
C19	ave(20-22)	1.35	1.32	0.03	1.28	0.07
C12	ave(17-19)	2.47	2.24	0.23	2.38	0.09
			**MAD:**	**0.11**	**MAD:**	**0.12**

*Literature reference for experimental data.

Deprotonated Compound **2** (Carboxylate):

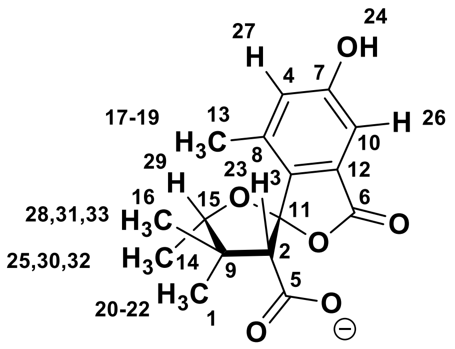

Energy profiles for the conformers found for deprotonated **2**. The percent population is shown, with those included in the calculation highlighted in green. Data is sorted by relative Gibb's free energy.

B3LYP/6-31G(d)		
	ΔG (kcal/mol)	% population
2.log	0.000	80.0
1.log	0.820	20.0

Computed chemical shifts (ppm) for compound deprotonated **2**. Values were obtained by scaling the calculated isotropic values of individual atoms, using the scaling factors noted in the method section. The chemical shifts shown are Boltzmann weighted average of the corresponding conformers. Mean absolute deviations (MADs) (between the computed and experimental numbers, ppm) maximum absolute deviations (MAXs) are also included.

^{13}C			Isolation[1]		Synthesis[2]	
**Expt. #s**	**Comp #**	**Comp.**	**Expt.**	**Abs. Dev.**	**Expt.**	**Abs. Dev.**
C17	C9	16.60	17.3	0.7	17.2	0.6
C1	C2	60.90	60.7	0.2	60.4	0.5
C15	C15	140.35	137.5	2.8	137.4	2.9
C18	C16	122.69	124.6	1.9	124.7	2.0
C19	C1	172.59	170.4	2.2	170.4	2.2
C13	C3	172.16	170.8	1.4	170.7	1.5
C11	C8	158.43	160.9	2.5	161.0	2.6
C4	C12	137.52	136.1	1.4	136.2	1.3
C10	C4	46.52	43.8	2.7	43.8	2.7
C8	C10	107.58	108.6	1.0	108.6	1.0
C9	C7	115.57	113.3	2.3	113.1	2.5
C14	C11	131.65	131.0	0.6	131.0	0.6
C2	C5	17.27	17.6	0.3	17.6	0.3
C3	C6	14.91	15.0	0.1	14.9	0.0
C12	C13	85.52	87.5	2.0	87.4	1.9
C16	C14	25.64	26.2	0.6	26.1	0.5
			**MAD:**	**1.4**	**MAD:**	**1.4**

*Literature reference for experimental data.

^{1}H			Isolation[1]		Synthesis[2]	
**Expt. #s**	**Comp #**	**Comp.**	**Expt.**	**Abs. Dev.**	**Expt.**	**Abs. Dev.**
C1	H23	3.54	3.55	0.01	3.51	0.03
C15	H29	4.24	4.24	0.00	4.21	0.03
C10	H27	7.11	6.97	0.14	6.95	0.16
C8	H26	7.09	6.99	0.10	6.93	0.16
-	H24	5.18	n.o.		n.o.	
C16	H34	1.32	1.32	0.00	1.28	0.04
C18	ave(25,30,32)	1.46	1.38	0.08	1.36	0.10
C19	ave(28,31,33)	1.39	1.32	0.07	1.28	0.11
C12	ave(20-22)	2.48	2.24	0.24	2.38	0.10
	ave(17-19)		**MAD:**	**0.08**	**MAD:**	**0.09**

*Literature reference for experimental data.

Full Gaussian References

Gaussian 09, Revision B.01,

M. J. Frisch, G. W. Trucks, H. B. Schlegel, G. E. Scuseria, M. A. Robb, J. R. Cheeseman, G. Scalmani, V. Barone, B. Mennucci, G. A. Petersson, H. Nakatsuji, M. Caricato, X. Li, H. P. Hratchian, A. F. Izmaylov, J. Bloino, G. Zheng, J. L. Sonnenberg, M. Hada, M. Ehara, K. Toyota, R. Fukuda, J. Hasegawa, M. Ishida, T. Nakajima, Y. Honda, O. Kitao, H. Nakai, T. Vreven, J. A. Montgomery, Jr., J. E. Peralta, F. Ogliaro, M. Bearpark, J. J. Heyd, E. Brothers, K. N. Kudin, V. N. Staroverov, T. Keith, R. Kobayashi, J. Normand, K. Raghavachari, A. Rendell, J. C. Burant, S. S. Iyengar, J. Tomasi, M. Cossi, N. Rega, J. M. Millam, M. Klene, J. E. Knox, J. B. Cross, V. Bakken, C. Adamo, J. Jaramillo, R. Gomperts, R. E. Stratmann, O. Yazyev, A. J. Austin, R. Cammi, C. Pomelli, J. W. Ochterski, R. L. Martin, K. Morokuma, V. G. Zakrzewski, G. A. Voth, P. Salvador, J. J. Dannenberg, S. Dapprich, A. D. Daniels, O. Farkas, J. B. Foresman, J. V. Ortiz, J. Cioslowski, and D. J. Fox, Gaussian, Inc., Wallingford CT, 2010.

Gaussian 09, Revision D.01,

M. J. Frisch, G. W. Trucks, H. B. Schlegel, G. E. Scuseria, M. A. Robb, J. R. Cheeseman, G. Scalmani, V. Barone, B. Mennucci, G. A. Petersson, H. Nakatsuji, M. Caricato, X. Li, H. P. Hratchian, A. F. Izmaylov, J. Bloino, G. Zheng, J. L. Sonnenberg, M. Hada, M. Ehara, K. Toyota, R. Fukuda, J. Hasegawa, M. Ishida, T. Nakajima, Y. Honda, O. Kitao, H. Nakai, T. Vreven, J. A. Montgomery, Jr., J. E. Peralta, F. Ogliaro, M. Bearpark, J. J. Heyd, E. Brothers, K. N. Kudin, V. N. Staroverov, T. Keith, R. Kobayashi, J. Normand, K. Raghavachari, A. Rendell, J. C. Burant, S. S. Iyengar, J. Tomasi, M. Cossi, N. Rega, J. M. Millam, M.

Klene, J. E. Knox, J. B. Cross, V. Bakken, C. Adamo, J. Jaramillo, R. Gomperts, R. E. Stratmann, O. Yazyev, A. J. Austin, R. Cammi, C. Pomelli, J. W. Ochterski, R. L. Martin, K. Morokuma, V. G. Zakrzewski, G. A. Voth, P. Salvador, J. J. Dannenberg, S. Dapprich, A. D. Daniels, O. Farkas, J. B. Foresman, J. V. Ortiz, J. Cioslowski, and D. J. Fox, Gaussian, Inc., Wallingford CT, 2013.

1. Elsebai, M. F.; Nazir, M.; Kehraus, S.; Egereva, E.; Ioset, K. N.; Marcourt, L.; Jeannerat, D.; Gütschow, M.; Wolfender, J.-L.; König, G. M., Polyketide Skeletons from the Marine Alga-Derived Fungus Coniothyrium cereale. *European Journal of Organic Chemistry* **2012,** *2012* (31), 6197-6203.
2. Ren, Z.; Hao, Y.; Hu, X., Total Synthesis and Structural Reassignment of (±)-Cereoanhydride. *Organic Letters* **2016,** *18* (19), 4958-4961.
3. Hagen, R.; Roberts, J. D., Nuclear magnetic resonance spectroscopy. Carbon-13 spectra of aliphatic carboxylic acids and carboxylate anions. *Journal of the American Chemical Society* **1969,** *91* (16), 4504-4506.
4. Gottlieb, H. E.; Kotlyar, V.; Nudelman, A., NMR Chemical Shifts of Common Laboratory Solvents as Trace Impurities. *The Journal of Organic Chemistry* **1997,** *62* (21), 7512-7515.
5. Hua-Jian Xu, Yong-Qiang Zhao, and Xin-Feng Zhou, Palladium-Catalyzed Heck Reaction of Aryl Chlorides under Mild Conditions Promoted by Organic Ionic Bases, *Journal of Organic Chemistry*, **2011**, *76* (19), pp 8036–8041.
6. Schah-Mohammedi, P.; Shenderovich, I. G.; Detering, C.; Limbach, H.-H.; Tolstoy, P. M.; Smirnov, S. N.; Denisov, G. S.; Golubev, N. S., Hydrogen/Deuterium-Isotope Effects on NMR Chemical Shifts and Symmetry of Homoconjugated Hydrogen-Bonded Ions in Polar Solution. *Journal of the American Chemical Society* **2000,** *122* (51), 12878-12879.
7. Spectral Database for Organic Compounds (SDBS); NMR spectrum; SDBS No.: 1033HSP-03-549; http://riodb01.ibase.aist.go.jp/sdbs/
8. Spectral Database for Organic Compounds (SDBS); NMR spectrum; SDBS No.: 610HPM-00-375; http://riodb01.ibase.aist.go.jp/sdbs/
9. Lodewyk, M. W.; Siebert, M. R.; Tantillo, D. J., Computational Prediction of 1H and 13C Chemical Shifts: A Useful Tool for Natural Product, Mechanistic, and Synthetic Organic Chemistry. *Chemical Reviews* **2012,** *112* (3), 1839-1862.
10. Tantillo, D. J., Walking in the woods with quantum chemistry - applications of quantum chemical calculations in natural products research. *Natural Product Reports* **2013,** *30* (8), 1079-1086.
11. Di Micco, S.; Chini, M. G.; Riccio, R.; Bifulco, G., Quantum Mechanical Calculation of NMR Parameters in the Stereostructural Determination of Natural Products. *European Journal of Organic Chemistry* **2010,** *2010* (8), 1411-1434.
12. Grimblat, N.; Sarotti, A. M., Computational Chemistry to the Rescue: Modern Toolboxes for the Assignment of Complex Molecules by GIAO NMR Calculations. *Chemistry – A European Journal* **2016,** *22* (35), 12246-12261.
13. Willoughby, P. H.; Jansma, M. J.; Hoye, T. R., A guide to small-molecule structure assignment through computation of (1H and 13C) NMR chemical shifts. *Nat. Protocols* **2014,** *9* (3), 643-660.
